# Treating steroid-dependent psoriasis from the blood-heat syndrome perspective: A case report

**DOI:** 10.1097/MD.0000000000045926

**Published:** 2025-11-07

**Authors:** Mei-Yang Jiang, Yong Jiang

**Affiliations:** a School of Basic Medicine, Chengdu University of Traditional Chinese Medicine, Chengdu City, China.

**Keywords:** blood, cool blood and resolve toxins, dependent psoriasis, heat syndrome, steroid

## Abstract

**Rationale:**

Psoriasis is a chronic, inflammatory dermatosis with a complex pathogenesis. Steroid-dependent psoriasis represents a therapeutic challenge, as conventional treatments often yield suboptimal responses and relapse. In traditional Chinese medicine (TCM), psoriasis is categorized as “Bai Bi,” and the blood-heat syndrome is a core pathological pattern driving inflammation and progression. However, the efficacy of TCM strategies specifically targeting this syndrome in steroid-dependent cases requires further documentation.

**Patient concerns:**

A 68-year-old male presented with a 20-year history of recurrent, widespread erythematosquamous plaques, and severe, refractory pruritus. The condition had acutely exacerbated in the preceding week. Previous long-term therapies with topical corticosteroids and calcipotriol provided only transient improvement with frequent recurrence.

**Diagnoses:**

The patient was diagnosed with active-phase, steroid-dependent psoriasis (Western medicine). TCM syndrome differentiation identified a pattern of blood-heat with dampness-wind. Retrospective severity assessment estimated a psoriasis area and severity index score of 16.7 and body surface area involvement of 15%, indicating severe disease.

**Interventions:**

A stage-adapted TCM therapeutic strategy was employed, centered on a modified “Buffalo Horn and Rehmannia Decoction” (Shui Jiao Dihuang Tang). The core principle was to cool the blood, resolve toxins, and dispel dampness and wind. The prescription was dynamically adjusted across 3 clinical visits over 3 months based on symptom evolution, including dose modifications of key herbs like Buffalo Horn and the addition of agents to address pruritus, sleep disturbance, and residual hyperpigmentation.

**Outcomes:**

Following the 3-month intervention, complete resolution of skin lesions and elimination of pruritus were achieved. The patient maintained clinical remission without recurrence during an 11-month follow-up period, based on self-report.

**Lessons:**

This case documents clinical improvement following TCM intervention in steroid-dependent psoriasis. Important limitations include the single-case design, lack of control group, retrospectively estimated psoriasis area and severity index/body surface area scores, and self-reported long-term outcomes. The observed temporal association between intervention and improvement may reflect natural disease variation, placebo effects, or other confounding factors. While this preliminary observation merits further investigation through rigorous prospective studies, the current evidence level remains limited and cautions against causal interpretation.

## 1. Introduction

Psoriasis manifests as erythematous plaques with silvery scaling, pruritus, and pain, ranging from localized to generalized involvement.^[1]^ Its pathogenesis involves genetic, immunological, and environmental interactions.^[2]^ Global prevalence reaches 2% to 3% with a significant comorbidity burden.^[1,2]^ First-line therapies (topical corticosteroids, vitamin D analogs, and biologics)^[3]^ show limited efficacy in corticosteroid-dependent cases, often yielding suboptimal responses and relapse.

In traditional Chinese medicine (TCM), psoriasis is termed “Bai Bi.” TCM serves as a primary alternative therapy for patients with prolonged symptoms (stubborn pruritus, erythematous scales, and exudate) or steroid dependence. Compared to conventional Western treatment, TCM offers advantages including lower recurrence rates and better compatibility with adjuvant therapies. Clinical evidence confirms its efficacy in improving quality of life and resolving lesions.^[4]^ Professor Jiang Yong therapeutic protocol based on the blood-heat syndrome perspective demonstrates significant clinical efficacy in treating psoriasis.

## 2. Patient information

Patient: Mr. Liu, Male, 68 years old, Han Chinese, Retired (Businessman).

Chief complaint: Recurrent scaly rash over the entire body accompanied by severe pruritus for 20 years, with acute exacerbation in the past week.

Present illness: Twenty years ago, erythematous scaly skin lesions appeared diffusely over the body without identifiable precipitating factors. Previous topical treatments with corticosteroids and calcipotriol proved ineffective.

Past medical history: Denies history of hypertension, diabetes mellitus, or hyperlipidemia.

Family history: No documented family history of psoriasis.

Psychosocial impact: Significant pruritus causing sleep disturbance.

Treatment history: No documented family history of psoriasis.

Topical therapy: Application of corticosteroid/calcipotriol ointments resulted in temporary lesion improvement but with frequent recurrence.

Multiple treatment attempts across various institutions failed to achieve persistent remission.

## 3. Clinical findings

### 3.1. Physical examination (initial consultation)

Cutaneous manifestations: Widespread erythematous papules, desquamation, and scaling; lesions are prominent on limbs, neck, and back.

Signs: Reddish discoloration, yellowish exudate, scratch marks, and crusting.

Systemic symptoms: Oral stickiness, anorexia, fatigue, heaviness sensation, and sticky stools with incomplete evacuation.

Tongue/pulse: Red tongue with yellow greasy coating; slippery, and rapid pulse. (In TCM diagnosis, these tongue and pulse signs suggest the presence of heat and dampness pathogens in the body).

## 4. Severity assessment

To provide a quantitative estimation of psoriasis severity, we retrospectively estimated the psoriasis area and severity index (PASI) and body surface area (BSA) based on clinical photographs and documentation from the initial consultation. It should be noted that these scores were not prospectively collected, which may affect their accuracy and is also a common limitation in retrospective case reports. The PASI score evaluates the severity of erythema, infiltration, and desquamation (each scored 0–4) as well as the percentage of affected area across 4 body regions, with a total score ranging from 0 to 72. BSA estimates the percentage of body surface area covered by lesions. At baseline, the patient’s condition was estimated as severe, with a PASI score of approximately 16.7 and a BSA of 15%.

## 5. Diagnostic assessment

Diagnostic methods: Clinical diagnosis based on characteristic lesions (erythematosquamous plaques, scaling) and chronic history. TCM syndrome differentiation: Blood-heat syndrome with dampness-heat accumulation (complicating factor of fluid metabolism dysfunction and exudation).

Diagnostic challenges: Long-term steroid dependence masks typical progression; no histopathology/biomarker confirmation.

Diagnostic reasoning: Differential diagnoses: Eczema, cutaneous T-cell lymphoma (excluded by chronicity and treatment response).

Confirmed diagnosis: Active-phase psoriasis (western medicine); Blood-heat syndrome with dampness-wind (indicating inflammation with exudation and itching).

Prognostic characteristics: Severe chronicity (20-year history) and steroid dependence indicated poor prognosis; TCM intervention achieved full remission.

## 6. Therapeutic intervention

A stage-adapted treatment strategy was employed: Core therapy: Buffalo Horn and Rehmannia Decoction Adjuvant intervention: Strict dietary avoidance of spicy/pungent foods to eliminate sources of dampness-heat transformation.

Decoction and administration protocol: Buffalo horn powder was wrapped separately and decocted for 40 minutes before adding other herbs.

Each dose was administered over 2 days, 3 times daily (150 mL per serving) after meals. It is important to note that this dosing regimen means the daily intake of each herb was effectively half of the total amount per formulaCoix seed (Yi Yi Ren, Coicis Semen) and Germinated Barley (Mai Ya, Hordei Fructus Germinatus were consistently included to protect stomach function against cold herb toxicity.

**Figure 1.**
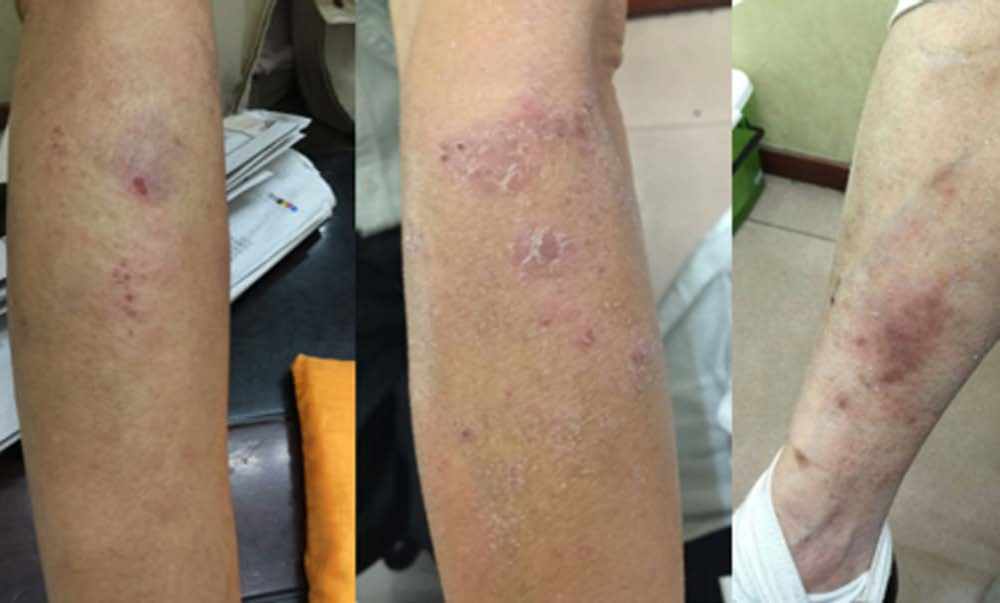
Skin condition after treatment.

Dynamic intervention adjustments: First visit (October 14, 2024, active phase) Figure 1.

Syndrome characteristics: Blood-heat with dampness-toxin accumulation (erythematous exudative lesions, red tongue with yellow greasy coating).

Therapeutic principle: Cool blood (reduce inflammation at the blood level) and resolve toxins as the primary approach (clear pathogenic factors), clear heat and dry dampness (reduce heat and dry exudation) as the secondary support.

Prescription highlights:

Sovereign herb: Buffalo horn powder 60 g to aggressively clear blood-level heat-toxin.

Minister herbs: Arnebia root (Zi Cao, *Arnebiae Radix* 30 g to activate blood circulation and resolve macules (improve blood microcirculation and resolve lesions); Kochia fruit (Di Fu Zi, Kochiae Fructus 45 g to dispel wind and relieve itching (alleviate itching).

Adjuvants: Gypsum 60 g and Spreading Hedyotis Herb (Bai Hua She She Cao, Hedyotis Diffusae Herba)” 30 g to enhance qi-level dampness-heat clearance.

Second visit (October 25, 2024, transition phase).

Syndrome evolution: Reduced heat-toxin but lingering spirit agitation (sleep disturbance and irritability, manifested as decreased pruritus but persistent sleep disturbance and anal burning).

Prescription modifications: Buffalo horn powder was reduced to 40 g to prevent excessive cooling.

New additions: Fried Spiny Jujube Seed (Suan Zao Ren, Ziziphi Spinosae Semen 30 g to nourish the heart (nourish the heart to calm the mind for sleep) and calm the spirit (clear heart fire to reduce irritability).

Coptis root (Huang Lian, *Coptidis Rhizoma*) 15 g to clear heart fire and relieve vexation.

Removal of’ dragon bone (Long Gu, Mastodi Ossis Fossilia) to avoid qi stagnation (prevent disruption of energy flow).

**Figure 2.**
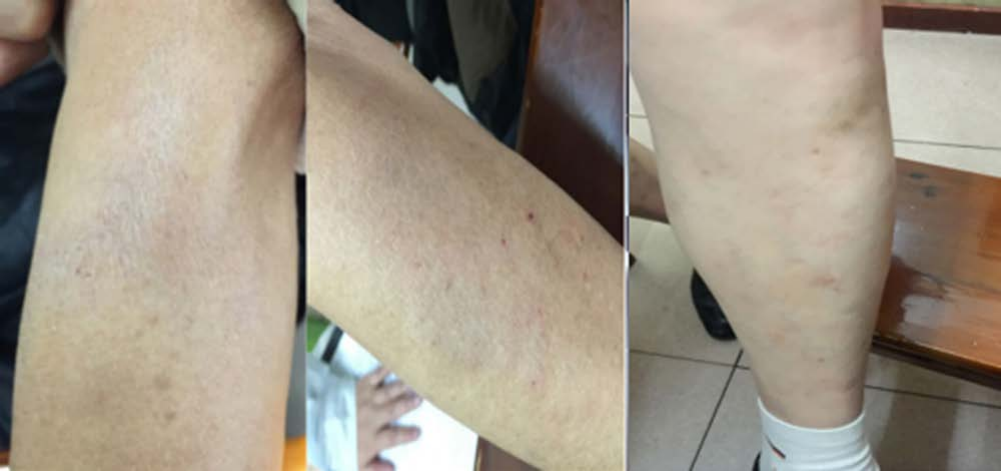
Physical findings at the third clinical visit (November 25, 2024).

Third visit (November 25, 2024, resolution phase) Figure 2.

Key manifestations: Residual pathogen in collaterals (hyperpigmentation on lower limbs) and unrecovered fluids (dry stools).

Therapeutic shift: Inclusion of insect-derived herb: Black-tail Snake (Wu Shao She, Zaocys 20 g to expel wind and unblock collaterals (disperse pathogenic wind and unblock circulation in chronic lesions), resolving chronic stasis.

Fluid-nourishing and spleen-fortifying agents: Chinese Yam (Shan Yao, Dioscoreae Rhizoma) 30 g, and Dendrobium stem (Shi Hu, Dendrobii Caulis) 20 g to reinforce earth (disperse pathogenic wind and unblock circulation in chronic lesions) and generate fluids.

Buffalo horn powder readjusted to 60 g (paired with Ophiopogon root (Mai Dong, Ophiopogonis Radix 30 g to counteract dryness), preventing heat-toxin recurrence.

## 7. Follow-up and outcomes

Clinician-assessed outcomes: Visit 2 (day 11): >80% lesion regression; pruritus resolved; exudation ceased. Visit 3 (day 42): Complete clinical resolution; stable without recurrence (Fig. 2).

Follow-up test results: Not applicable (clinical monitoring only). Adherence and Tolerability: Adherence: Full compliance reported; no missed doses. Tolerability: No adverse events; mild perianal discomfort (resolved by visit 3). Adverse Events: None reported.

Long-term follow-up: To further assess the durability of remission, the patient was recontacted via telephone in September 2025. He self-reported sustained complete remission without any recurrence of psoriatic lesions or pruritus, representing a total follow-up period of approximately 11 months (from October 2024 to September 2025). While encouraging, this long-term outcome is based on patient self-report without contemporaneous clinical confirmation. This sustained remission was temporally associated with the preceding treatment intervention; however, the design of this study precludes attributing it definitively to the TCM treatment (Table 1).

**Table 1:** Timeline of clinical events.

Date	Event	Phase	PASI score (estimated)	BSA (%) (estimated)	VAS
October 14, 2014	Acute exacerbation: active plaques ± exudate	Active	16.7	15%	9
October 10, 2024	Pruritus ↓; exudate resolved	Transitional	5.1	5%	2
November 25, 2024	Lesions cleared; residual hyperpigmentation	Remission	<1.0	<1%	0
September 20, 2025	No recurrence reported; sustained remission	Long-term follow-up	<1.0	<1%	0

BSA = body surface area, PASI = psoriasis area and severity index, VAS = visual analog scale.

## 8. Discussion

Strengths and limitations in the management of this case. The principal strength of this management lies in achieving complete resolution of steroid-dependent psoriasis refractory to conventional treatments through rigorous TCM syndrome differentiation. Key merits include the successful reversal of a 20-year disease chronicity within just 3 months, alongside sustained remission during follow-up without recurrence. The approach further demonstrated clinical value through individualized formula modifications that specifically addressed concurrent dampness-heat manifestations such as sticky stools and yellow, greasy coating. Notably, the strategic incorporation of insect-derived medicinals, including centipede and black-tail snake, proved essential for effective collateral unblocking in this chronic presentation.

However, several limitations must be acknowledged. The single-case design (n = 1) inherently restricts the generalizability of the findings. Major methodological limitations include the absence of a control group and the reliance on retrospectively estimated rather than prospectively collected objective severity scores (PASI, BSA). Furthermore, the use of high-dose herbs, such as Buffalo Horn powder at 60 g per formula, warrants scrutiny.Therefore, the temporal association between the observed clinical improvement and the herbal intervention, while suggestive of a potential treatment response, does not establish causality. Natural disease fluctuation or other unmeasured confounding factors could also have played a role.

It should be emphasized that the safety of this aggressive prescription was carefully managed under the TCM compatibility principle of “sovereign (Jun), minister (Chen), assistant (Zuo), and envoy (Shi)”—a structured framework aimed at enhancing efficacy and reducing toxicity through herb synergism. Specifically, the sovereign herb Buffalo Horn was used at twice the conventional dose to vigorously clear heat-toxin from the blood level, an approach supported by studies indicating its dose-dependent effect and short-term safety in acute severe psoriasis.^[4]^ Minister herbs such as Arnebia root (30 g/formula), employed to activate blood circulation and resolve macules, also exceeded common dosage ranges. This strategy is grounded in clinical literature showing that combination therapy can attenuate potential hepatotoxicity in refractory blood-heat presentations.^[5]^ Similarly, the assistant herb Coptis root (15 g/formula) was applied at a high dose to clear heart fire, with evidence supporting its short-term use in recalcitrant damp-heat conditions.^[6]^ To counteract risks associated with such an intensive regimen, other assistant and envoy herbs played essential roles: Gypsum cleared heat at the qi level, while stomach-protecting agents like Coix seed and Germinated barley harmonized the middle energizer and moderated the cold nature of the principal herbs. Additionally, the drying tendency of Coptis was balanced by incorporating moistening agents such as Ophiopogon root. Clinically, this comprehensive strategy resulted in no reported adverse events and sustained remission.

Notwithstanding these deliberate precautions within the TCM paradigm, the safety and tolerability of high-dose herbal formulations in wider patient populations remain uncertain and warrant further validation through controlled studies. This concern is further heightened by the absence of long-term immunological monitoring, which precludes mechanistic insight into the treatment response.

Psoriasis is a chronic inflammatory dermatosis affecting 2% to 3% globally,^[7,8]^ characterized by erythematous plaques with silvery scaling^[7]^ and significant comorbidity burden.^[8]^ Its pathogenesis involves complex genetic-immunological interactions.^[9,10]^ Conventional first-line therapies (corticosteroids, vitamin D analogs, and biologics)^[11]^ exhibit critical limitations: corticosteroid dependence/atrophy,^[7]^ hypercalcemia risk from vitamin D analogs, and infection/cost burdens with biologics.^[11,12]^ TCM approaches targeting blood-heat syndrome demonstrate complementary value through multi-mechanistic actions—cooling blood, resolving toxins, dispelling wind—with documented efficacy in improving lesions and quality of life.^[13,14]^ Current evidence validates heat-clearing and blood-cooling herbs (e.g., Buffalo Horn, Rehmannia) as core therapeutic strategies,^[10,13,14]^ aligning with our intervention.

Based on the case assessment, the primary pathogenic mechanism underlying this steroid-dependent psoriasis was identified as blood-heat syndrome compounded by dampness-heat toxicity, leading to fluid consumption, wind generation, and cutaneous stagnation. The key therapeutic rationale centered on stage-adapted intervention through cooling blood, resolving toxins, clearing heat, and dispelling dampness/wind. Modified Buffalo Horn and Rehmannia Decoction served as the core prescription, dynamically adjusted to address evolving symptoms: Initial high-dose buffalo horn (60 g) with heat-clearing/toxin-resolving herbs (e.g., honeysuckle, forsythia) rapidly controlled acute inflammation. Insect-derived agents (centipede, black-tail snake) were later incorporated to unblock collaterals in chronicity. Adjuncts targeted comorbidities: wind-dispelling herbs (Dictamni bark) for pruritus, yin-nourishing agents (ophiopogon) for scaling-related dryness, and sedatives (Jujube Seed) for sleep disruption.

## 9. Experience

This case presents a novel therapeutic paradigm for managing long-term, steroid-dependent psoriasis through a TCM approach that precisely targets the “blood-heat syndrome.” Utilizing a modified *Buffalo Horn and Rehmannia Decoction* (Shui Jiao Dihuang Tang) with stage-adapted modifications to address comorbid dampness, pruritus, and residual hyperpigmentation led to complete lesion resolution and sustained remission. This outcome underscores the significant potential of rigorous TCM syndrome differentiation as an effective strategy for challenging psoriasis cases refractory to conventional treatments.

## 10. Limitations

The conclusions of this study are tempered by several important limitations inherent in its design. As a retrospective case report of a single patient (n = 1), the generalizability of the findings is severely limited. Most importantly, the study design cannot infer causality; the observed association between clinical improvement and the therapeutic intervention should be considered preliminary and might be influenced by confounding factors, the natural course of the disease, or placebo effects. The absence of a control group, the use of retrospectively estimated (rather than prospectively collected) objective outcome measures (e.g., PASI, BSA), and the lack of immunological data (e.g., cytokine levels) constrain the objective assessment of efficacy and the understanding of underlying mechanisms. Furthermore, the long-term follow-up outcome was based on patient self-report without clinical reassessment. Additionally, while the high-dose herbal regimen (e.g., Buffalo Horn at 60 g per formula) was managed within the TCM compatibility framework and no adverse events were observed, its safety profile requires systematic evaluation in larger, controlled populations.

## 11. Future perspectives

Building upon the limitations identified, future research should aim to validate these promising findings through rigorously designed randomized controlled trials that include appropriate control groups and prospective, objective metrics. Mechanistic studies incorporating serial immunological profiling (e.g., of Th17-related cytokines) are essential to scientifically elucidate how the TCM principle of “cooling blood and resolving toxins” modulates inflammatory pathways in psoriasis. Concurrently, pharmacological and safety studies are warranted to systematically assess the pharmacokinetics and tolerability of high-dose herbal prescriptions, such as the 1 described here, to ensure their safe integration into evidence-based clinical practice.

## 12. Patient perspective

The patient with steroid-dependent psoriasis reported profound suffering from intractable itching (especially nocturnal), widespread scaling plaques disrupting sleep, and associated fatigue/digestive discomfort. Following TCM treatment, he experienced complete resolution of skin lesions, elimination of pruritus, restored sleep quality, and sustained remission with no recurrence during follow-up, expressing high treatment satisfaction.

## Acknowledgments

We thank the efforts and contributions of the reported patients and all the clinical staff in this study.

## Author contributions

**Writing – original draft:** Mei-Yang Jiang.

**Writing – review & editing:** Yong Jiang.
